# Primary Tooth Decay Prevention Program in Children: Application of Intervention Mapping Approach

**DOI:** 10.1155/2022/8901102

**Published:** 2022-04-14

**Authors:** Bahareh Kabiri, Alireza Heidarnia, Mehdi Mirzaei Alavijeh, Mohammad Esmaeel Motlagh

**Affiliations:** ^1^Health Education and Health Promotion, Non-Communicable Diseases Research Center, Ilam University of Medical Sciences, Ilam, Iran; ^2^Department of Health Education and Health Promotion, Faculty of medical Sciences, Tarbiat Modares University, Tehran, Iran; ^3^Social Development & Health Promotion Research Center, Health Institute, Kermanshah University of Medical Sciences, Kermanshah, Iran; ^4^School of Medicine, Jundishapur University of Medical Sciences, Ahvaz, Iran

## Abstract

**Background:**

As the most common diseases globally, oral and dental diseases are closely related to people's behavior. The present study is aimed at developing a program to prevent primary teeth decay in children using the intervention mapping approach.

**Methods:**

This study protocol is aimed at using the intervention mapping approach as the planning framework. The study consists of six steps of intervention mapping, including needs assessment based on the relevant literature review, development of an objectives matrix for changing people's behaviors and environmental factors, program preparation, program implementation, and program evaluation to develop a family-centered program.

**Results:**

The use of the intervention mapping approach helped us identify the outcomes and functional objectives, develop an appropriate intervention program, and evaluate the program.

**Conclusions:**

The intervention mapping approach is an appropriate guide to developing a systematic and evidence-based program.

## 1. Background

Oral health plays a key role in the overall health of the body [1, 2]. The prevalence of dental caries has increased progressively among children and adolescents over the past two decades in developing countries, and, despite many advances in oral health, it is still considered a health problem in developing and even developed countries [3, 4]. Tooth decay in young children has a significant impact on their health and social and intellectual development and is associated with complications such as pain, eating problems, and high financial costs for the family and society [5–8].

Tooth decay is a global disease, and few populations are immune to its effects [9]. It affects 60%-90% of young people and most adults worldwide and often leads to pain and discomfort [10]. In developed countries, the dental caries reduction program begins in childhood [9].

Families are advised to supervise their children's toothbrushing with fluoride toothpaste for up to 7 years to prevent tooth decay in the UK. 31% of five-year-old children have dental caries in this country, increasing to more than 41% in deprived areas [11].

In this regard, communities with no prevention program have poor healthcare systems and/or are at a low level of socioeconomic status, general health, and standard of living [9]. The value of the decayed, missing, and filled teeth (DMFT) index in six-year-old children in Iran is 5.84, of which 85.14% is related to the number of decayed deciduous teeth. The corresponding numbers are 6.73 and 89%, respectively, in Ilam Province [12].

Parents are advised to supervise their children's toothbrushing process from about six months to 7 years of age; this can lead to a 15% reduction in tooth decay [7, 12, 14].[13] Most parents ignore attention to their children's deciduous teeth and only take them to a dentist when they have severe and unbearable pain or dental abscess-induced face swelling. There is no choice but to extract the primary teeth [15].

According to some studies, fear, anxiety, psychological problems and low self-efficacy, parental education levels, number of children, family economic status, and awareness of the importance of toothbrushing can affect oral health behaviors [16–18].

Some studies have focused on behavioral change at the individual level, but toothbrushing in children under the supervision of parents is an interpersonal behavior influenced by a wide range of factors [19, 20]. Given the high prevalence of oral and dental diseases in Iran [12] and the limited number of family-based programs in Iranian society, it seems necessary to use a planning approach in which the health-related problems, based on the problem-solving perspective and the ecological approach. This planning approach should help adapt the interventions to the new population and environments as much as possible and provide a classification of behavior change techniques for developing the contents of the interventions [21]. The present study is aimed at developing a program to prevent primary teeth decay in children using the intervention mapping approach.

## 2. Methods

The present study was conducted to develop a family-centered program using the intervention mapping approach. As described by Bartholomew et al., the intervention mapping protocol provides a framework for developing, implementing, and evaluating health promotion programs [22] and is known as a successful and effective protocol for creating health-based intervention programs [23, 24].

The protocol has six steps. The first step pertains to needs assessment, the second to fourth steps include the initial development of the intervention, the fifth step consists of planning for implementation, and the sixth step includes the evaluation and modification of the intervention. Activities in each mapping step will guide the following step [25, 26].

### 2.1. Step 1: Needs Assessment

The intervention mapping approach defines the pathway of identifying the needs or the health problems that determine the solution [27].

In the first step, before starting to plan the program, the problem of health, quality of life, and the behavioral and environmental causes of the problem, and their determinants were addressed using the preceding model [21]. This step included two parts: (1) the epidemiological-behavioral analysis and the social analysis of the group or community at risk and its problems and (2) attempts at recognizing and beginning to understand the characteristics of the community, its members, the capacity of the community, and its knowledge of the health problem and its possible solutions. The planning group of the family-centered research included health education and health promotion professionals, pediatricians, pediatric dentists, and community members who used the preceding model to assess the needs. The program's strengths included the cooperation and participation of the Comprehensive Urban Health Center No. 6 and the interest of the mothers in learning the materials for their children. The program's weaknesses included the poor participation of the working mothers since they were busy working during the group discussion hours and participated in the program when no group discussions were held.

Consequently, individual discussion and consultation sessions were held for the working mothers. The results obtained from the review of the relevant literature indicated that the individual (the mothers) and the environmental (organizational and social) levels were the program's most important ecological levels. The preliminary study of the needs assessment group on the causes of health problems showed that some factors such as fear and anxiety, psychological problems and low self-efficacy levels of the individuals, fatigue, laziness and impatience, depression, lack of time and high cost of living, parental education level, mother's occupation, birth order, number of children, family economic status, awareness, attitude, and health literacy of the mothers affected oral health behaviors [15–17, 28–31].

Mothers and environmental behaviors were categorized into 8 and 3 behaviors, respectively. In addition, the cognitive determinants related to individual and environmental behaviors such as awareness, attitude, perceived severity, perceived benefits and perceived barriers, cues to action, perceived self-efficacy, behavioral intention, subjective norms, and social norms were also identified (Tables 1 and 2).

### 2.2. Step 2: Identification of Intervention Targets

The second step of intervention mapping pertains to creating a change objectives matrix as the main tool of intervention mapping. This step examines the importance and possibility of behavior changeability [32].

Functional objectives are specified in this step. These objectives guide the health intervention program and are expected effects of the health intervention program. The most important part of the functional objectives is what the patients need to do to turn the desired behavior into a habit.

The change objectives in the intervention program reflect the changes needed in sociocognitive determinants for achieving the functional objectives. Therefore, each functional objective may include several change objectives [24].

In other words, the second step is aimed at focusing on changing individual behavior and the environmental actors' behavior. The draft of the functional objectives based on the theory of planned behavior (TPB, health belief) was first drawn up by the workgroup preparing the matrix based on the individual and interpersonal outcomes of the program. Then, a 10-member group of pediatricians, dentists, and health educators was asked to validate the draft. After receiving their views, the functional objectives were revised. Finally, after writing the change objectives and creating individual and environmental level matrices, the logical pattern of program change was completed (Tables 1 and 2).

### 2.3. Step 3: Selection of Theory-Based Intervention Methods and Applications

The third step of intervention mapping included the creation of program ideas, modification methods, and practical applications [33]. In this step, while creating the program ideas in the planning group, the theoretical methods influencing the changes in the determinants were investigated, and the items related to the process evaluation were created.

### 2.4. Step 4: Integration of Methods and Applications into an Organized Programmed

The program was developed in the fourth step of the intervention mapping. Steps 1 to 3 completed the fourth step. The end product of this step was a comprehensive plan for the intervention program. This plan helped the program designers develop creative materials for supporting health education and health promotion programs. In this step, the pretest was carried out, the practical applications were experimentally implemented, and program materials were developed with the participation of the recipients and the implementers. In this task, the planning team and the design workgroup focused on the cultural relevance of the program since planning that lacks a cultural basis will jeopardize the project. In this step, attention was paid to all the cultural aspects, whether superstructure or infrastructure, in the community. They were accurately identified, and attempts were made to utilize them in designing the program positively. Since being continuously in touch with the participants is a method to enhance adaptation of the program to the culture, close attention was paid to interpersonal channels.

Several ideas must be considered to find a good one [34].

Brainstorming is the most appropriate way of getting ideas from the group members. The program's field, sequence, and components, which constitute its units, were then determined to provide 12 hours of training in 4 weeks in the family-centered program.

Due to the role of face-to-face training and its advantage over delivering lectures, and in order to attract the participation of the mothers faced with lack of time (due to having children under the age of one), 2 groups of 15 members were formed in this program, and 12 hours of training were divided between the two groups. The training was then presented to them in a completely identical manner. In addition, the local language was used in the training sessions to make the program more effective.

In the family-centered program, the graphic designer participated and the printing house and the available films in the Department of Oral and Dental Health in the Ministry of Health and Medical Education were used.

The main documents used in this study included educational pamphlets (to increase awareness in and provide action guide for the mothers), educational clips (to increase the perceived intensity of the mothers), replicas (to increase the mothers' awareness), lectures and group discussions (to enhance awareness, attitude, perceived intensity, perceived benefits, perceived barriers, subjective norms, and social norms), and modeling (to increase perceived self-efficacy). The initial design of the pamphlet was prepared by the research team and finalized by the graphic designer and then the printing house, and the educational films on oral health behaviors were prepared by the Department of Oral and Dental Health which were shown to the mothers. The replicas in the Dental Department of Ilam University of Medical Sciences were also used. In addition, four phone calls per month were made to the mothers to augment the action guides for them (Table 3).

### 2.5. Step 5: Plan for Adoption, Implementation, and Sustainability

The program's implementation plan was the fifth step of the intervention mapping. In this step, the potential implementers of the program were identified, the outcomes and the functional objectives of the program were stated, the matrices of change objectives were created to be used in the program, and the implementation interventions were designed. In this step, two 2-hour meetings were held between the planner, the health educators, and the head of the Comprehensive Urban Health Center No. 6 to review program adoption, implementation, and sustainability to coordinate their actions as much as possible.

The activities during this step provided a basis for evaluating the efficiency and effectiveness of the study so that the matrices provided in Step 2 were used to develop evaluation scales for effectiveness the matrices developed in Step 5 to develop the questions and the process evaluation scales.

### 2.6. Step 6: Planning the Evaluation Program

This research assessed the determinants of oral health behaviors in the mothers for program evaluation.

The program's effectiveness was evaluated using a randomized controlled trial (RCT) (IRCT20190716044227N1). This study was conducted from May to December 2019 with the approval of the Ethics Committee at the Faculty of Medical Sciences in Tarbiat Modares University with the ethics code IR.MODARES.REC.1398.02.

According to previous studies, with type I error of 5%, and the power of the study of 95%, the sample size was calculated to be 60 subjects. To this end, 60 mothers with children aged six months to one year were randomly selected among those visiting the Comprehensive Urban Health Center No. 6 in Ilam City (western Iran). They were then randomly divided into the two 30-member intervention and control controls.

### 2.7. Inclusion and Exclusion Criteria

The inclusion criteria were having a healthy child of 6 months to one year and a health record and being literate. Women who were pregnant women or had an underlying disease were excluded from the research.

Ilam City is divided into Districts 1 and 2 according to city divisions. District 1 has 9 Comprehensive Health Centers and 3 Urban Health Bases, and District 2 has 2 Comprehensive Health Centers and 4 Urban Health Bases. The total population of District 1 is 110,099, of which 55,141 are female. The Comprehensive Urban Health Center No. 6 included the largest population among the urban centers and, as the only educational center in the province, had suitable educational space. Therefore, it was selected for implementing the training program. In this stage, the mothers were selected using simple random sampling from those invited to participate in the study at the Comprehensive Urban Health Center No. 6. The participants were then randomly allocated to the intervention and control groups. The data were collected using a written questionnaire at baseline and 6 months after the end of the program.

### 2.8. Data Analysis

The collected data were analyzed in SPSS 12. The independent and the paired-sample *t*-tests were used to evaluate the program's effectiveness. The chi-squared test evaluated the two groups in terms of the underlying variables and the studied determinants.

## 3. Results

Before implementing the training program, the intervention and control groups were examined for demographic variables. No statistically significant differences were observed between them in this regard (Table 4). The two groups were also evaluated using the independent-sample *t*-test regarding the study determinants before implementing the training program. No statistically significant differences were found between the two groups in this respect either.  Table 5 shows the mean coefficients of variation in the determinants of oral health behavior. Based on these results, the intervention program improved the intention to adopt oral health behaviors.

## 4. Discussion

The present study is aimed at developing and evaluating a program to prevent primary tooth decay in children based on the intervention mapping protocol in Ilam Province located in western Iran. Our literature review showed no published study in Iran on developing oral and dental interventions using IM. This randomized controlled trial showed the usefulness of the intervention program in improving the intention to adopt oral and dental hygiene behaviors in mothers. Our findings showed a significant increase in the mean awareness score after the training intervention. The educational strategy used to improve the awareness level of mothers included lectures, group discussions, educational pamphlets, and the use of replicas to familiarize the participants with the structures of the mouth and teeth. Consistent with the present research results, the findings of various studies have shown an increase in the score for awareness after training interventions [35–37].

The mean score for attitude increased in this research in the intervention group after the training intervention through group discussions and lectures. This result agrees with those of the study by Bourke in Australia [38].

McGowan proposed the balanced decision-making method as the most appropriate technique for improving the attitude of patients with diabetes or cancer. In this method, patients are encouraged to list the advantages and disadvantages of behavior change and make short- and long-term decisions [39].

Another result of the present study was a significant increase in the mean score for perceived self-efficacy in the mothers after implementing the training program. This increase can be attributed to the employed educational strategies as modeling, verbally encouraging performance of minor functions, and expressing successful experiences by the participants were used in this research.

Self-efficacy is an important predictor of compliance. People who show the most change usually have higher self-efficacy in performing certain behaviors [40].

The present study's findings show a significant increase in the mean score for perceived benefits, perceived intensity, and cues to action after implementing the training program. The training strategy included making four phone calls to the mothers per month, lectures, and group discussion. Similar studies reported that discussion was the most effective method in increasing perceived intensity [41].

According to the findings, the mean behavioral intention of the mothers increased significantly six months after the end of the program. Modeling was used as the training strategy for improving the intention of adopting oral health behavior. To this end, the mothers of the children with white and healthy teeth were invited to share their experiences with the other mothers.

Achterberg et al. used the operational planning method as a suitable theoretical technique for changing the sociocognitive determinant of intention. The operational planning method is aimed at changing behaviors, i.e., when patients intend to make a plan to change their behavior, operational planning focuses on creating new behaviors similar to self-management in patients with chronic health problems. Operational planning can include changing the existing behavior or stopping unwanted behavior [42].

Another finding of the present study was the significant decrease in the mean score for the perceived barriers in the mothers of the experimental group compared to the control group. These changes may indicate the effectiveness of the training program on the mothers' perceived barriers in improving their intention to adopt oral health behaviors.

The training strategies used to reduce perceived barriers included lectures and group discussions. The simplicity of toothbrushing and using appropriate methods for breastfeeding infants were stated, the possible barriers to each of these behaviors were discussed, and the opportunity for group discussion was provided. These strategies eventually led to overcoming barriers such as lack of time, fatigue, and boredom.

Lack of time was one of the major barriers that were mentioned in relation to observing oral health. Extensive training can minimize this barrier because upgrading lifestyle requires focusing on the quality of life improvement [43].

Some studies have shown that coping planning effectively changes the sociocognitive determinant of barriers. The use of this method requires the determination of high-risk situations. High-risk conditions can stop the patient from changing the behavior to the desired one even if this change has been planned. In addition to determining the high-risk situations, learning to correctly cope with such situations is necessary. Coping planning focuses on the sociocognitive determinants of barriers and barrier control skills and mentions the need to continue behavior change. In practice, those patients change their behavior that encounters barriers to behavior change. Coping planning makes the patient feel slightly uncomfortable in behavior change [44].

Another study result was the high mean score for the mothers' subjective norms in the experimental group compared to the control group. The employed training strategy for improving the subjective norms included using the modeling method, employing group discussion, and evoking the inner feelings and a sense of responsibility in the mothers regarding their children's adoption of oral health behaviors.

Ebrahimipour et al. conducted a study entitled “*Investigating the effect of a training program based on the theory of planned behavior on improving oral health behavior in pregnant women visiting urban health bases in Ashkhaneh City in Iran*.” The results showed that the training intervention was not effective on the abstract norms of the women. In addition, there was a significant difference between the intervention and control groups in this regard after the training intervention. This finding, which is inconsistent with the present study results, can be attributed to the lack of participation of a large number of spouses in the training sessions, insufficient training for the companions, and low quality of the training intervention [45].

The present study's findings showed a significant increase in the mean score for oral norms related to oral health in the mothers of the experimental group compared to the control group. These changes indicate the effectiveness of the training program in improving the social norms related to oral health in mothers. Using the method of showing educational films was the training strategy used to improve the subjective norms.

In a comparative study conducted in the Caribbean and Nepal entitled “*Factors determining and upgrading oral hygiene behaviors: a study based on the theory of planned behavior*,” both groups of the participants in the Caribbean and Nepal emphasized the impact of social norms on adopting oral health behaviors. The Caribbean participants reported no social pressure on them to adopt oral health behaviors. They felt that good control and self-management could effectively develop oral health behaviors [46].

Coster and Norman acknowledged in their studies that informing patients without any support (e.g., self-management) was not effective and did not influence their health [47].

This research had some strengths and weaknesses. In relation to its strengths, it was the first clinical trial performed on Kurdish-speaking Iranian mothers to improve oral health behaviors. In addition, considering the children's age, the intervention was performed at the interpersonal and environmental (organizational and social) levels and focused on the mothers. As for its weaknesses, it was probably accompanied by some errors because a questionnaire was used as the research instrument. It was also time-consuming due to the nature of using intervention mapping in designing interventions. As reported by other researchers who used the intervention mapping protocol, this study showed that intervention mapping, while being complex and time-consuming, was flexible and could greatly help improve the program's effectiveness [48–50]. The small sample size was a limitation in this study that may affect the results and should be resolved in the future.

## 5. Conclusions

The present study provided comprehensive information on developing interventions to improve oral health behavior. It also forms the cornerstone for implementing similar programs in different parts of Iran and even in other countries.

## Figures and Tables

**Table 1:** Examples of functional objectives and interpersonal change objectives for the improvement of oral health behavior.

Determinants	Self-efficacy	Knowledge	Attitude	Intensity	Perceived benefits	Perceived barriers	Behavioral intention	Subjective norm
Direct behaviors of mothers effective in preventing premature decay of deciduous teeth in children: 1: mothers should perform oral hygiene practices for their children to learn.								
Change objectives	Functional objectives							
Mothers should perform oral hygiene practices for their children to learn.	1.1.2. Mothers should express their confidence in cleaning their children's teeth and gums.	1.2.2. Mothers should be aware of oral health behaviors and prevent premature tooth decay in their children.	1.2.3. Mothers should express their belief in the prevention of premature tooth decay by observing the oral hygiene practices for their children.	1.2.4. Mothers should recognize that their children are at risk for premature decay if they do not perform oral hygiene practices.	1.2.5. Mothers should explain the benefits of oral hygiene to their children.	1.2.6. Mothers should be aware of the barriers to oral health for their children.	1.2.7. Mothers should express their intention to have their children perform oral health behaviors.	1.2.8. Mothers should reward themselves for having their children perform oral hygiene practices.
Mothers should perform oral hygiene practices for their children to learn.	1.2.1. Mothers should guarantee that they will take their time to clean their children's teeth.							
Mothers should perform oral hygiene practices for their children to learn.	1.3.1. Mothers should guarantee that they will take their children to the dentist for dental examinations.							
Mothers should perform oral hygiene practices for their children to learn.	1.4.1. Mothers should guarantee not to put milk bottles in their children's mouths at night or for long periods of time.							
Mothers should perform oral hygiene practices for their children to learn.	1.5.1. Mothers should guarantee not to breastfeed their children for long periods of time.							
Mothers should perform oral hygiene practices for their children to learn.	1.6.1. Mothers should not give sugar-containing materials to their children frequently as snacks.							
Mothers should perform oral hygiene practices for their children to learn.	1.7.1. Mothers should not put sweet liquids in their babies' mouths for long periods of time.							
Mothers should perform oral hygiene practices for their children to learn.	1.8.1. Mothers should clean their children's gums and teeth after feeding them milk, sweet liquids, and snacks.							

**Table 2:** Examples of functional objectives and change objectives at the organizational and social levels for the improvement of Oral Health Behavior.

Determinants	Social norm	Action guides
Indirect behaviors Outcome 1: mothers should perform oral hygiene behaviors for their children to learn.		
Functional objectives	Change objectives	
Society avoids accepting our children as long as their teeth are decayed and smell bad.	Mothers should state that, given their awareness of society's view on oral health, they are successful in preventing tooth decay in their children.	
Dentists support mothers in having their children perform oral health behaviors.		Mothers should state that they are successful in preventing dental caries in their children with the support and guidance of dentists.
Spouses support mothers in having their children perform oral health behaviors.		Mothers should state that they are successful in preventing dental caries in their children with the support of their spouses.

**Table 3:** A review of the determinants, methods, and parameters used in the family-centered program to improve oral health behaviors.

Determinant	Method	Parameters used for measurements
Awareness	Group discussion, pamphlets, lectures, and replicas	Questionnaire
Attitude	Lecture and group discussion	Questionnaire
Perceived intensity	Group discussion and educational clips	Questionnaire
Perceived benefits	Lecture and group discussion	Questionnaire
Perceived barriers	Lecture and group discussion	Questionnaire
Action guide	Phone calls and pamphlets	Questionnaire
Self-efficacy	Successful experiences, verbal encouragement, and modeling	Questionnaire
Behavioral intention	Modeling	Questionnaire
Subjective norm	Discussion and modeling	Questionnaire
Social norm	Film shows	Questionnaire

**Table 4:** Comparison of intervention and control groups in terms of demographic variables before implementation of the training program.

Variable		Group		Significance level
		Number (%)	Number (%)	
Father's educational level	Not finished high school	3 (5)	4 (6.7)	0.86
	High school diploma	12 (20)	14 (23.3)	
	University degrees	15 (25)	12 (20)	
Mother's educational level	Not finished high school	8 (13.3)	7 (11.7)	0.84
	High school diploma	15 (25)	15 (25)	
	University degrees	7 (11.7)	8 (13.3)	
Father's occupation	Retired	—	1 (1.7)	0.14
	Unemployed	4 (6.7)	4 (6.7)	
	Employed	26 (41.4)	25 (41.7)	
Mother's occupation	Housewife	18 (30)	19 (31.7)	0.53
	Employed	12 (20)	11 (18.4)	

**Table 5:** The mean coefficient of variation of the oral health behavior determinants in the intervention and control groups before and after the training intervention.

Determinant	Group	Before intervention (mean ± SD)	After intervention (mean ± SD)	*P* value
Awareness	Intervention group	13.03 ± 3.2	22.3 ± 1.5	0.001
	Control group	12.93 ± 3.05	12 ± 2.6	0.87
Attitude	Intervention group	17.90 ± 2.3	21.13 ± 1.3	0.001
	Control group	17.40 ± 2.4	16.86 ± 3.2	0.20
Perceived intensity	Intervention group	11.06 ± 2.3	15.76 ± 1.6	0.001
	Control group	11.40 ± 2.12	11.50 ± 2.5	0.63
Perceived benefits	Intervention group	12.10 ± 2.2	15.83 ± 1.7	0.001
	Control group	12.30 ± 2.5	12.53 ± 2.3	0.39
Perceived barriers	Intervention group	12.43 ± 2.5	8.73 ± 2.1	0.001
	Control group	12.93 ± 2.5	12.76 ± 2.3	0.15
Action guide	Intervention group	9.96 ± 1.71	12.20 ± 1.18	0.003
	Control group	9.86 ± 1.43	10.10 ± 1.32	0.39
Self-efficacy	Intervention group	17.40 ± 2.9	20.23 ± 2.6	0.002
	Control group	17.06 ± 3.3	16.33 ± 2.9	0.09
Behavioral intention	Intervention group	19.03 ± 1.8	20.43 ± 1.8	0.003
	Control group	18.50 ± 2.2	17.96 ± 1.9	0.13
Subjective norm	Intervention group	13.70 ± 2.2	16.20 ± 1.4	0.001
	Control group	13.50 ± 2.1	13.90 ± 1.7	0.19
Social norm	Intervention group	14.36 ± 2.5	16.33 ± 1.7	0.001
	Control group	14.03 ± 2.8	83.13 ± 2.3	0.40

## Data Availability

All data generated or analyzed during this study are included in this published article.
